# Supplementary material for: The Impact of TSC-1 and -2 Mutations on Response to Therapy in Malignant PEComa: A Multicenter Retrospective Analysis
Source: Genes (Basel). 2022 Oct 24;13(11):1932. doi: 10.3390/genes13111932 (PMC9689779; doi:10.3390/genes13111932)
Supplement: Supplementary file 1 [file genes-13-01932-s001.zip › Table S1.pdf]

genes

MDPI

Supplementary Section

Table S1. Patient Information.

ID	Pathologic Diagnosis	Malignant PEComa*	Surgical Resection	Metastatic at Diagnosis	Local Recurrence	Firstline Treatment	Response	Time to Progression (months)	Time to Death (months)
WU-1	PEComa	No	Yes	No	No	N/A	N/A	N/A	218.04
WU-2	PEComa	No	Yes	No	No	N/A	N/A	N/A	69.24
WU-3	PEComa	No	Yes	No	No	N/A	N/A	N/A	4.9
WU-4	PEComa	No	Yes	No	No	N/A	N/A	N/A	1.02
WU-5	PEComa	Yes	No	Yes	Unknown	N/A	N/A	N/A	1.22
WU-6	PEComa	Yes	Yes	Yes	Yes	Paclitaxel	Unknown	2.79	5.33
WU-7	PEComa	No	Yes	No	No	N/A	N/A	N/A	133.97
WU-8	Angiomyolipoma	No	Yes	No	Yes	mTOR Inhibitor	Partial	54.35	119.47
WU-9	Angiomyolipoma	No	Yes	No	No	N/A	N/A	N/A	103.1
WU-10	Angiomyolipoma	No	Yes	No	No	N/A	N/A	N/A	1.84

WU-11	PEComa	No	Yes	No	No	N/A	N/A	N/A	0.2
WU-12	Angiomyolipoma	Yes	Yes	Unknown	Unknown	Everolimus	No evidence of disease (resected)	32.84	32.84
WU-13	Angiomyolipoma	No	Yes	Unknown	No	N/A	N/A	N/A	0.36
WU-14	Unknown	No	Yes	Unknown	No	N/A	N/A	N/A	0.85
WU-15	Angiomyolipoma	Yes	Yes	Yes	Yes	Sirolimus	Partial	9.90	24.07
WU-16	Unknown	No	Yes	No	No	N/A	N/A	N/A	57.01
WU-17	PEComa	No	Yes	No	No	N/A	N/A	N/A	45.73
WU-18	PEComa	No	Yes	No	No	N/A	N/A	N/A	48.89
WU-19	PEComa	Yes	Yes	Yes	No	ABI-009	Stable Disease	3.00	47.08
WU-20	Angiomyolipoma	Yes	Yes	Yes	Unknown	Everolimus	Stable Disease	71.70	94.55
WU-21	Lyphangioleiomyomatosis	No	Yes	No	Yes	ABI-009	Partial	39.30	43.2

WU-22	PEComa	No	Yes	No	No	N/A	N/A	N/A	36.53
WU-23	PEComa	Yes	Yes	No	No	N/A	N/A	N/A	29.29
WU-24	PEComa	No	Yes	No	No	N/A	N/A	N/A	29.59
WU-25	PEComa	Yes	Yes	Yes	No	Pembrolizumab	Partial	20.22	42.12
WU-26	PEComa	No	Yes	No	No	N/A	N/A	N/A	17.29
WU-27	PEComa	Yes	Yes	Yes	Unknown	Everolimus	Unknown	1.45	3.29
WU-28	PEComa	Yes	Yes	No	No	N/A	N/A	N/A	14.83
WU-29	Angiomyolipoma	No	Yes	No	No	N/A	N/A	N/A	12.46
WI-30	PEComa	Yes	Yes	Yes	Yes	Temsirolimus	Progressive Disease	2.00	24.76
WI-31	PEComa	Yes	Yes	No	No	N/A	N/A	N/A	4.6
IO-32	PEComa	No	Yes	No	No	N/A	N/A	N/A	0
IO-33	PEComa	No	Yes	No	No	N/A	N/A	N/A	93.8

IO-34	PEComa	Yes	Yes	No	No	Sirolimus	No evidence of disease (resected)	99.58	99.58
IO-35	PEComa	Yes	Yes	Yes	Yes	Everolimus	Unknown	8.15	8.15
IO-36	PEComa	Yes	Yes	Yes	Yes	Sirolimus	Partial	14.04	14.04
IO-37	PEComa	Yes	Yes	No	Yes	Everolimus	Stable Disease	35.01	35.01
IO-38	PEComa	Yes	Yes	No	No	N/A	N/A	N/A	12.23
39	PEComa	Yes	Yes	No	Yes	Doxorubicin and Ifosfamide	Unknown	109.32	188.58
40	PEComa	Yes	Yes	Yes	Yes	Gemcitabine and Docetaxel	Stable Disease	4.87	14.76
VU41	Unknown	Yes	Yes	No	No	N/A	N/A	N/A	15.75
VU42	PEComa	Yes	Yes	Yes	Yes	Doxorubicin and Olaratumab	Progressive Disease	1.00	9.4

VU43	PEComa	Yes	Yes	Yes	Yes	Sirolimus	Stable Disease	3.22	5.52
VU44	PEComa	Yes	Yes	No	No	N/A	N/A	N/A	2.56
VU45	Unknown	No	Yes	No	No	N/A	N/A	N/A	23.38
VU46	Angiomyolipoma	Yes	Yes	Yes	Yes	Doxorubicin and Dacarbazine	Progressive Disease	2.66	95.64
47	PEComa	Yes	No	Yes	Yes	Sirolimus	Unknown	1.00	0.95
48	PEComa	Yes	No	Yes	Yes	Arimidex	Stable Disease	18.15	28.21
WU-30	Angiomyolipoma	Yes	Yes	Unknown	Unknown	N/A	N/A	N/A	0.5589

*Malignant PEComa was defined as pathology demonstrating two or more of the following: primary tumor > 5 cm, infiltrative, high nuclear grade and cellularity, mitotic rate ≥ 1/50 high power field, necrosis, vascular invasion.
