# Supplementary figures and images for: The Impact of TSC-1 and -2 Mutations on Response to Therapy in Malignant PEComa: A Multicenter Retrospective Analysis
Source: Genes (Basel). 2022 Oct 24;13(11):1932. doi: 10.3390/genes13111932 (PMC9689779; doi:10.3390/genes13111932)

Supplementary Section

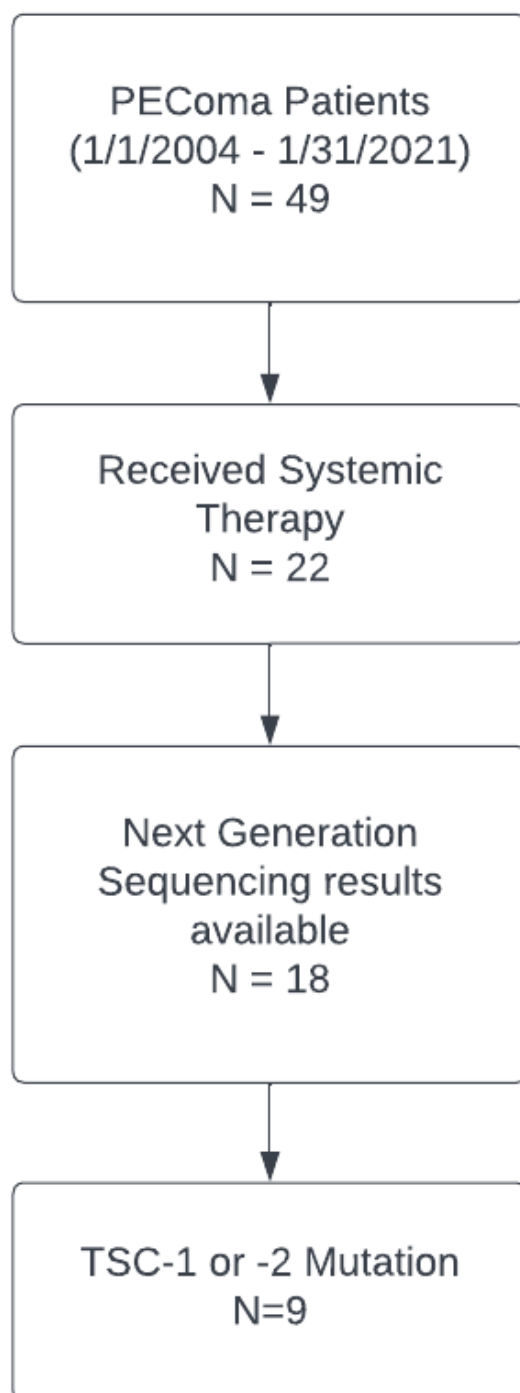

Figure S1. Flow Diagram.

Supplement: Supplementary file 1 [file genes-13-01932-s001.zip › Figure S1.pdf]
